# Aging Relevant Metabolite Itaconate Inhibits Inflammatory Bone Loss

**DOI:** 10.3389/fendo.2022.885879

**Published:** 2022-07-22

**Authors:** Yuting Wang, Song Li, Liming Zhao, Peng Cheng, Jian Liu, Fengjing Guo, Jun Xiao, Wentao Zhu, Anmin Chen

**Affiliations:** Department of Orthopaedic Surgery, Tongji Hospital, Tongji Medical College, Huazhong University of Science and Technology, Wuhan, China

**Keywords:** osteoporosis, aging, inflammation, itaconate, osteoclast

## Abstract

Progressive bone loss during aging makes osteoporosis one of the most common and life impacting conditions in geriatric populations. The bone homeostasis is maintained through persistent remodeling mediated by bone-forming osteoblast and bone-resorbing osteoclast. Inflammaging, a condition characterized by increased pro-inflammatory markers in the blood and other tissues during aging, has been reported to be associated with skeletal stem/progenitor cell dysfunction, which will result in impaired bone formation. However, the role of age-related inflammation and metabolites in regulation of osteoclast remains largely unknown. In the present study, we observed dichotomous phenotypes of anti-inflammatory metabolite itaconate in responding to inflammaging. Itaconate is upregulated in macrophages during aging but has less reactivity in responding to RANKL stimulation in aged macrophages. We confirmed the inhibitory effect of itaconate in regulating osteoclast differentiation and activation, and further verified the rescue role of itaconate in lipopolysaccharides induced inflammatory bone loss animal model. Our findings revealed that itaconate is a crucial regulatory metabolite during inflammaging that inhibits osteoclast to maintain bone homeostasis.

## Introduction

Osteoporosis is a common age-related skeletal disorder characterized by decreased bone mineral density and bone micro-architectural change. These changes lead to bone strength decline and contribute to bone fractures which could be life-threatening for geriatric populations (1). Bone is an active tissue that undergoes persistent remodeling. The maintenance of bone homeostasis relies on the balance of osteoblast mediated bone forming and osteoclast mediated bone resorption (2). Osteoblasts derive from skeletal stem/progenitor cells, whereas osteoclasts derive from hematopoietic monocyte/macrophage lineages (3, 4). Due to age-related stemness loss and/or microenvironment change, the development and activity of osteoblast and osteoclast have been largely remodeled during aging, leading to progressive bone loss and onset of osteoporosis (5, 6).

Immune system serves as the most important protection of organisms, by resisting foreign pathogens and eliminating injured or senescent autologous cells to maintain the homeostasis of our body. Both innate and adaptive immune systems undergo remarkable changes during aging. The most outstanding feature of innate immune system change is low-grade stimulation at basal level, whereas immune incompetent when specific reaction is needed (7, 8). Inflammaging, an emerging concept describing chronic, sterile, and low-grade inflammation during aging, has been shown to contribute to the pathogenesis of various age-related diseases (9, 10). In the skeletal system, aging induced circulating pro-inflammatory factors impair bone regeneration *via* decreased skeletal stem/progenitor cell number and osteogenic function (6). The role of inflammaging in regulating bone-resorbing osteoclast remains largely unknown.

Macrophages lie at the frontline of immune response, play crucial roles in both innate and adaptive immune systems. Accumulation of pro-inflammatory macrophages and release of cytokines in tissue have significant contributions to the inflammaging process (11, 12). Metabolic reprogramming of tricarboxylic acid cycle from oxidative phosphorylation to glycolysis is a hallmark of macrophage activation, generating endogenous metabolites which regulate inflammatory response (13, 14). Itaconate has been identified as one of the most highly induced metabolites during macrophage activation, playing an inhibitory role in inflammation (15, 16). Bone resorbing osteoclasts are derived from monocyte/macrophage lineages and governed by pro-inflammatory cytokines (3). Itaconate may therefore be involved in regulating bone homeostasis especially bone resorption during aging.

Here, we investigated the metabolic remodeling of macrophages during aging, provided insights into the regulatory role of macrophage derived metabolite itaconate in osteoclastogenesis and lipopolysaccharides (LPS) induced inflammatory bone loss.

## Materials and Methods

### Reagents and Antibodies

Antibodies targeting NF-κB pathway (IKKβ, p-IKKα/β, IκBα, p-IκBα, P65)P, p-P65) and MAPK pathway (JNK, p-JNK, ERK, p-ERK, P38, p-P38) were purchased from Cell Signaling Technology (Danvers, MA, USA). Recombinant murine M-CSF (Macrophage Colony-Stimulating Factor) and RANKL (Receptor Activator of Nuclear factor Kappa-β Ligand) were purchased from PeproTech (Rocky Hill, NJ, USA). TRAP staining kit, dimethyl itaconate (DI), and other reagents were purchased from Sigma-Aldrich (St. Louis, MO, USA). Culture mediums and fetal bovine serum (FBS) were obtained from Thermo Fisher Gibco (Waltham, MA, USA).

### Osteoclast Differentiation and Function

Bone marrow derived macrophages (BMMs) were isolated as previously described (17). Briefly, femur and tibia from C57BL/6 mouse were carefully dissected and rinsed in cold PBS before flushing out the bone marrow with α-MEM media containing 30ng/ml recombinant mouse M-CSF at day 0. After being left in a 10-cm dish for 16h in a 37°C incubator supplied with 5% CO_2_, supernatants with unattached cells were transferred to a new 10cm dish at day 1 and cultured for 2 more days to collect attached BMMs before a media change at day 3. BMMs were harvested with trypsin and replated for *in vitro* experiments at day 4. BMMs isolated from 2-month-old mice were used as young BMMs, and BMMs isolated from 2-year-old mice were used as aged BMMs for *in vitro* experiments in this study.

For osteoclast differentiation, BMMs were digested and plated to 96-well plate at the density of 20,000 cells per well, complete α-MEM media containing 30 ng/ml recombinant mouse M-CSF and 100 ng/ml recombinant mouse RANKL were applied to cells with daily media change until the end point designed by experiments. RAW264.7 cell lines were cultured in complete DMEM media containing 75 ng/ml RANKL for differentiation.

Trap staining kit was purchased from Sigma and performed following the manufacturer’s protocol after being fixed by PFA for identification of mature osteoclasts. Multinuclear (≥3) cells with positive trap staining were considered as mature osteoclast.

F-actin-ring and pit formation assay were performed to analyze the function of mature osteoclast as previously described (17). BMMs were plated on collagen coated plate for primary differentiation with α-MEM media containing 30 ng/ml M-CSF and 100ng/ml RANKL for 6 days before digested with collagenase and replated in Corning osteo assay strip wells. Cells were cultured in α-MEM media containing M-CSF and RANKL with indicated treatments for 3 more days before fixed for immunofluorescent staining and pit analysis. F-actin-tracker green purchased from Invitrogen were applied to the wells and incubated in 25°C for 1h after 4% PFA fixation and followed 0.1% triton-x perforation. 5 times of PBS wash were performed to remove nonspecific binding of actin tracker green and followed by 5 min DAPI staining to label the nuclear. F-actin-ring were imaged with Nikon microscope, and total number per wells were counted regardless of size. The wells were then bleached to remove the cells for pit analysis by quantifying the resorted area in the well.

### Quantitative Real-Time PCR and Western Blotting

Cells for Quantitative real-time PCR analysis were lysed with Trizol Reagent purchased from Invitrogen and RNA were extracted following the manufacturer’s protocol. Then the extracted RNA was reversed with the RevertAid First Strand cDNA Synthesis Kit (Thermo Scientific, Waltham, MA, USA). cDNA was used for real-time-qPCR with KAPA SYBR FAST qPCR Kit Master Mix (Kapa Biosystems, Hallandale, FL, USA). The primers listed below were used in real-time PCR to detect indicated genes: GCGAACGCT-GCCACTCA and ATCCAGGCTTGGAAGGTC for mouse IRG1; CTCCCACTCTTCCACCTTCG and TTGCTGTAGCCGTATTCATT for mouse GAPDH; GAAGAAGACTCACCAGAAGCAG and TCCAGGTTATGGGCAGAGATT for mouse CTSK; TCCTTGCAATGTGGATGTTT and CGTCCTT-GAAGA0AATGCAGA for mouse MMP9; TCTTCCGAGTTCACATCCC and GACAGCACCATCTT-CTTCC for mouse NFATc1; GATGCCAGC-GACAAGAGGTT and CATACCAGGGGATGTTGCGAA for mouse TRAP. The relative mRNA levels of target genes were calculated by the 2^–ΔΔ^ *^C^* ^T^ method, with GAPDH as an internal control and normalized to the control group (18).

Cells for Western blotting were lysed in RIPA reagent from Invitrogen supplemented with protease and phosphatase inhibitor. Cell lysates were sonicated and spined at 12000 g for 30 min at 4°C to remove cell debris, then boiled with SDS sample buffer and used for western blotting following CST’s protocol. Antibodies listed above were used to detect target protein. Quantification of protein level was performed with Gel-Pro Analyzer software with at least 3 biological repeats.

### Animals and LPS Induced Inflammatory Bone Loss Model

The use of animals and design of animal experiments were approved by the Institutional Animal Care and Use Committee of Tongji Hospital. All animals were supplied by the University Laboratory Animal Center. LPS induced inflammatory bone loss models were used for the *in vivo* experiments. 24 8-week-old male C57BL/6 mice were randomly assigned to four groups with n=6 in each group: Sham+Veh, Sham+DI, LPS+Veh, LPS+DI. No blinding was used during the experiment. 5mg/kg LPS (in LPS groups) or equal amount of PBS (in Sham groups) were subcutaneously injected on the middle cranial suture at day1 and day4, 30 mg/kg Dimethyl itaconate (in DI groups) or equivalent volume of PBS (in Veh groups) were given daily from day2 to day11 by intraperitoneal injection. The percentage of resorption area were calculated based on reconstructed CT scanning, Trap positive area per section in paraffin slides were used to evaluated osteoclast activity.

### Micro-Computed Tomography (μCT) Imaging

The skulls of mice from animal experiments were scanned with the vivaCT40 μCT instrument (Scanco Medical, Bassersdorf, Switzerland), bone signals were obtained at 100kV and 98μA, with the resolution as 10.5μm. Three dimensional images were reconstituted and analyzed with the built-in software.

### RNA-Sequencing of BMMs

BMMs were cultured with α-MEM in the absence of FBS for 12 h, then changed to complete media supplied with RANKL (100 ng/mL) and DI (20μM) or vehicle for 24 hours. Cells for RNA-sequencing were lysed with Trizol Reagent and submitted to Shanghai Biotechnology Corporation for the following process. Briefly, total RNA was extracted and checked for a RIN number to inspect RNA integrity. Qualified total RNA was further purified by RNAClean XP and RNase-Free DNase Set. The libraries were prepared with VAHTS mRNA-seq v2 Library Prep Kit for Illumina. The libraries were then sequenced with pair-end protocol, average raw bases data was 6Gb.

### Statistical Analysis

All *in vitro* experiments were performed independently with at least three biological repeats, and the results are presented as means ± standard error of the mean (SEM). The animal study was performed under the ARRIVE guidelines. The sample size, randomization, blinding, outcome measures and statistical methods are described in Animals and LPS induced inflammatory bone loss model section. One-way-ANOVA followed by Tukey *post hoc* corrections were used for multigroup comparison. Two-tailed Student’s t test was used for comparisons between 2 groups. In all analyses, P < 0.05 was taken to indicate statistical significance.

## Results

### Aging Associated Osteoclast Activation and Metabolism Remodeling

**Figure 1:**
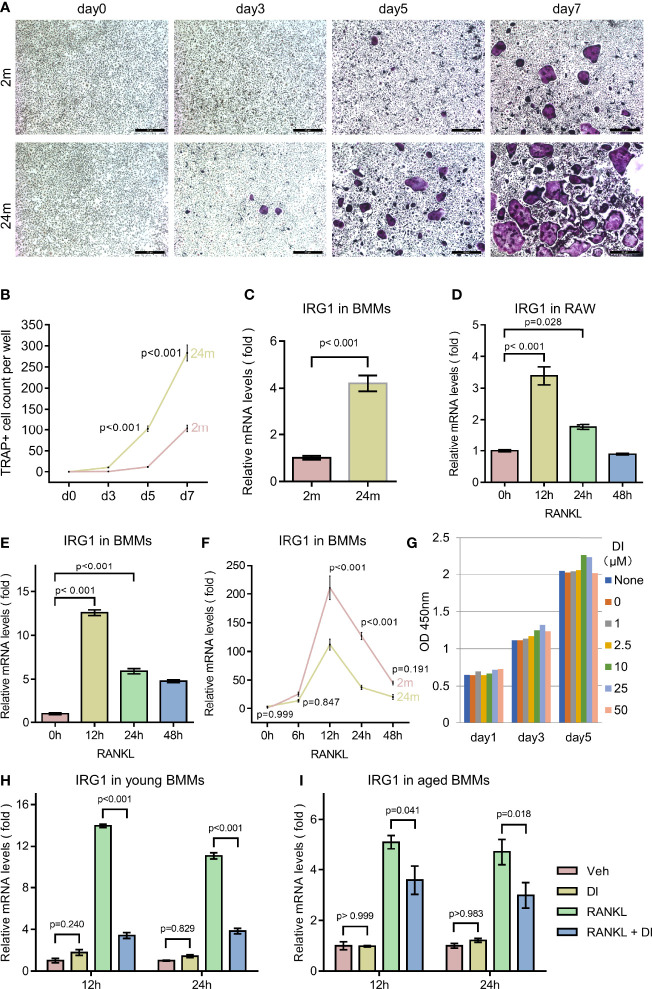
Aging associated osteoclast activation and metabolism remodeling. **(A, B)** BMMs isolated from 2-month-old mice (young) and 24-month-old mice (aged) were cultured with M-CSF and RANKL for the indicated time periods. TRAP staining was performed, and TRAP-positive cells with three or more nuclei were counted (scale bar = 50μm). **(C)** BMMs isolated from mice with indicated age were subjected to RNA extraction without any additional treatment, mRNA levels of IRG1 gene were assessed by q-PCR. **(D)** RAW264.7 cells were cultured in the presence of RANKL for indicated time before RNA extraction, mRNA levels of IRG1 were assessed by q-PCR. **(E)** BMMs isolated from 2-month-old mice were cultured with M-CSF and RANKL for the indicated hours before RNA extraction, mRNA levels of IRG1 were assessed by q-PCR. **(F)** BMMs isolated from young and aged mice were cultured with M-CSF and RANKL for indicated hours before RNA extraction, mRNA levels of IRG1 were assessed by q-PCR. **(G)** BMMs were plated 3000 cells per well with presence of M-CSF and treated with DI at indicated concentration, CCK8 assay were performed at day 1, 3, and 5. **(H, I)** The gene expression level of IRG1 in young **(H)** and aged BMMs **(I)** after DI treatment with or without RANKL was determined by qPCR. Data are presented as the mean ± SEM of three independent experiments with p value indicated in the figure.

Osteoporosis is an age-related bone disorder that could be due to decreased bone formation and/or increased bone resorption. Here in this study, we focused on osteoclast and its precursor macrophage during aging. In agreement with previous reports (19, 20), we observed an age-related increase of osteoclastogenesis activity between BMMs isolated from 2-month-old and 2-year-old C57BL/6 male mice in our *in vitro* experiments, indicated by faster osteoclast formation and more osteoclast formed (**Figures 1A, B**).

Aging has been shown to play an important role both in skewing the osteoclast precursor pool and promoting pro-inflammatory cytokine release in the bone marrow niches (20). While in response to inflammation, macrophage can also initiate metabolic reprogramming of the tricarboxylic acid cycle from oxidative phosphorylation to glycolysis to generate an anti-inflammatory function. To confirm the metabolic remodeling of macrophages during aging, we first test the IRG1/itaconate metabolic pathway. Itaconate is produced during metabolic remodeling, catalyzed by IRG1 encoded enzyme cis-Aconitate decarboxylase. IRG1 gene expression level has been shown to be an indicator of itaconate level (21). We compared IRG1 expression level between BMMs isolated from young and aged mice, observed a significantly increased IRG1 expression level in aged BMMs, indicating a metabolic remodeling status (**Figure 1C**).

Further we wonder if similar metabolic remodeling happens during RANKL induced osteoclastogenesis. We treated primary BMMs as well as macrophage cell line RAW264.7 with RANKL for different time periods. The IRG1 gene expression level increased rapidly to the peak level after 12 h RANKL treatment, then gradually decreased to basal level after 48h (**Figures 1D, E**). Interestingly, when comparing the response of IRG1 expression to RANKL stimulation between young and aged BMMs, we noticed that young BMMs generated higher IRG1 expression level at all time points tested (**Figure 1F**).

These results together suggested that BMMs undergo metabolic remodeling during aging and RANKL induced osteoclastogenesis, however the IRG1/itaconate metabolic pathway is more responsive in young BMMs. Therefore, we suspected that the different basal level and responsive level of the IRG1/itaconate pathway between young and aged BMMs may lead to distinct osteoclast phenotype. Next, we tested if the metabolic remodeling product itaconate plays a role in regulating osteoclastogenesis and activity.

### Itaconate Inhibits RANKL Induced Osteoclast Differentiation and Activation

To exclude the impact of itaconate on BMMs proliferation, we performed cell proliferation and cytotoxicity assay with Cell Counting Kit 8 (CCK8). Membrane permeable dimethyl itaconate (DI) was used in this study, as it has been shown to be the most potent itaconate derivate that stabilizes the anti-inflammatory transcription factor NRF2, inhibits IκBζ and pro-interleukin (IL)-1β induction, as well as IL-6, IL-10 and interferon-β secretion (22–24). In the presence of membrane permeable dimethyl itaconate (DI) up to 50μM, no reduction of cell counting was observed in BMMs (**Figure 1G**).

Several studies indicated that exogenous DI is insufficient to direct convert to endogenous itaconate. However, exogenous DI can increase endogenous itaconate accumulation in LPS stimulated macrophages (22, 25, 26). To test if exogenous DI can regulate the biosynthesis of endogenous itaconate, we performed qPCR for IRG1, the gene encoding enzyme cis-Aconitate decarboxylase which is responsible for itaconate biosynthesis. In resting BMMs, the IRG1 expression was not significantly affected by DI treatment, aligning with previous studies. Interestingly, upon RANKL stimulation, the IRG1 expression level was significantly downregulated by DI treatment in both young and aged BMMs (**Figures 1H, I**). So, it seems unlikely that DI can increase endogenous biosynthesis of itaconate through IRG1 expression in this case. If exogenous DI would be involved and occupy the endogenous itaconate degradation pathway, then prevent the degradation of endogenous itaconate remains unknown. But this could potentially explain the itaconate accumulation upon stimulation while not regulating the biosynthesis of itaconate.

**Figure 2:**
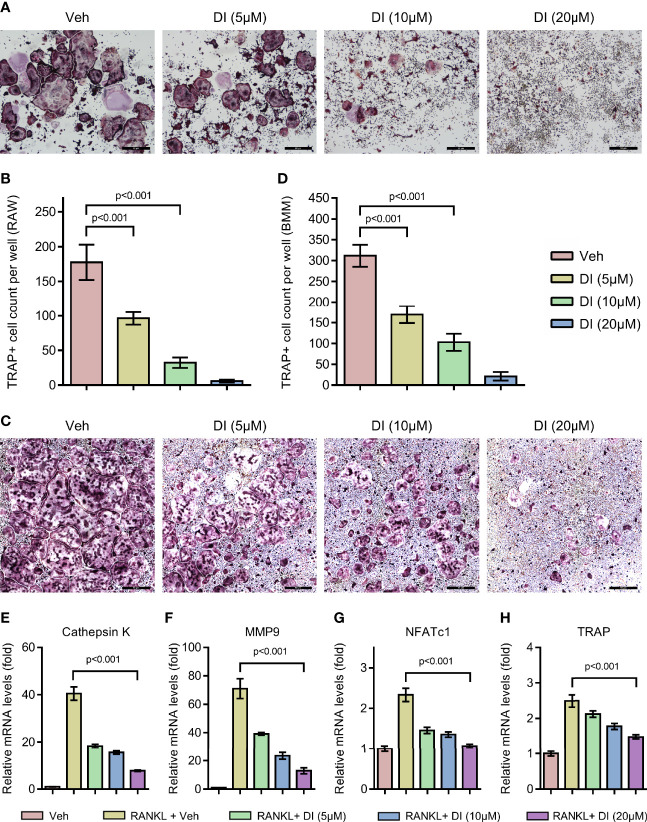
Itaconate inhibits RANKL induced osteoclast differentiation and activation. **(A, B)** RAW264.7 cells were seeded in 96-well plates overnight and then treated with different concentrations of DI or vehicle in the presence of RANKL for 4 days. TRAP staining was performed, and the TRAP-positive cells with three or more nuclei are counted in (scale bar = 50μm). **(C, D)** BMMs were cultured in the presence of M-CSF and RANKL and were treated with vehicle or DI with indicated concentration for 7 days. TRAP staining was performed, and TRAP- positive cells with three or more nuclei are counted in (scale bar = 50μm). **(E–H)** BMMs were treated with different concentrations of DI in the presence of M-CSF and RANKL for 3 days. The mRNA levels of osteoclast-related genes were assessed by qPCR. Data are presented as the mean ± SEM of three independent experiments with p value shown in the figure.

To investigate whether exogenous addition of itaconate could regulate osteoclast formation, we treated both macrophage cell line RAW264.7 and primary BMMs with itaconate during RANKL induced osteoclast differentiation. With TRAP staining, we observed that itaconate treatment significantly inhibited osteoclast differentiation in a dose dependent manner in both RAW264.7 and BMMs (**Figures 2A–D**). At the concentration of 20μM, osteoclast formation was totally blocked in the RAW264.7 cell line. The inhibitory effect of itaconate on osteoclast formation was further verified by q-PCR test showing that the mRNA levels of both osteoclast master transcription factor NFATc1 and osteoclast marker genes CTSK, MMP9 and TRAP were downregulated in the presence of itaconate in the same trend as TRAP staining (**Figures 2E–H**).

**Figure 3:**
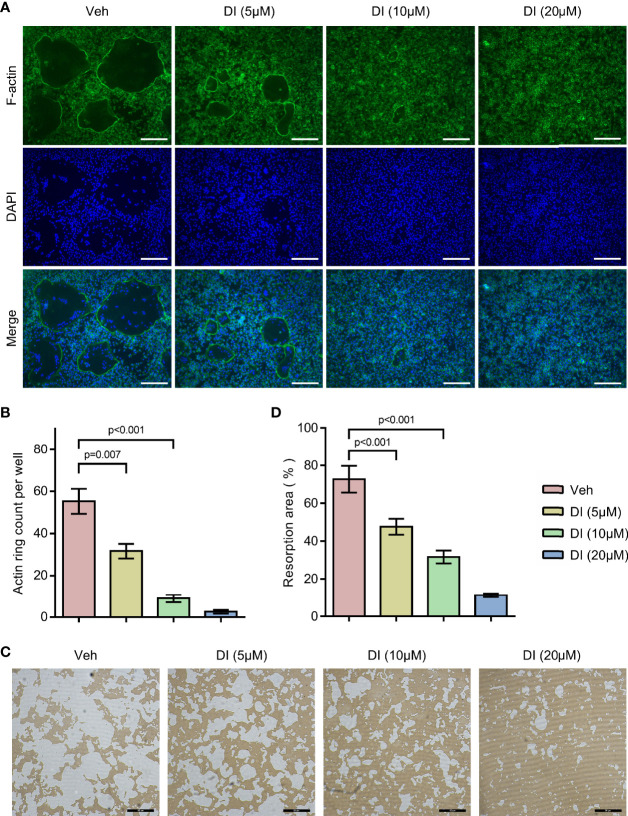
Itaconate inhibits the bone resorption activity of osteoclast. Mature osteoclasts from BMMs were seeded in Corning osteo assay strip wells, and they were treated with different concentrations of DI for 3 days in the presence of M-CSF and RANKL. **(A, B)** F-actin staining was performed. The number of actin rings was quantified (scale bar = 50μm). **(C, D)** Pit formation assays were performed. The resorption areas were quantified (scale bar = 50μm). Data are presented as the mean ± SEM of three independent experiments with p value shown in the figure.

To further study the potential effect of itaconate on osteoclast function, F-actin ring staining and bone resorption pit formation analysis were performed on matured osteoclast. BMMs were induced with RANKL to form matured osteoclasts, those osteoclasts were then plated into Corning osteo assay strip wells and treated with different concentrations of itaconate. F-actin ring staining results showed that the formation of F-actin ring was impaired under the treatment of itaconate at 5μM (**Figures 3A, B**). The quantification of the resorption pits showed that itaconate had a dose-dependent inhibitory effect on osteoclast resorption activity (**Figures 3C, D**).

The *in vitro* results revealed that aging or RANKL induced metabolite itaconate serves as a negative regulator of osteoclast differentiation and resorption activity. Further we tested if itaconate could prevent inflammatory bone loss *in vivo*.

### Itaconate Attenuates LPS Induced Inflammatory Bone Loss *In Vivo*

**Figure 4:**
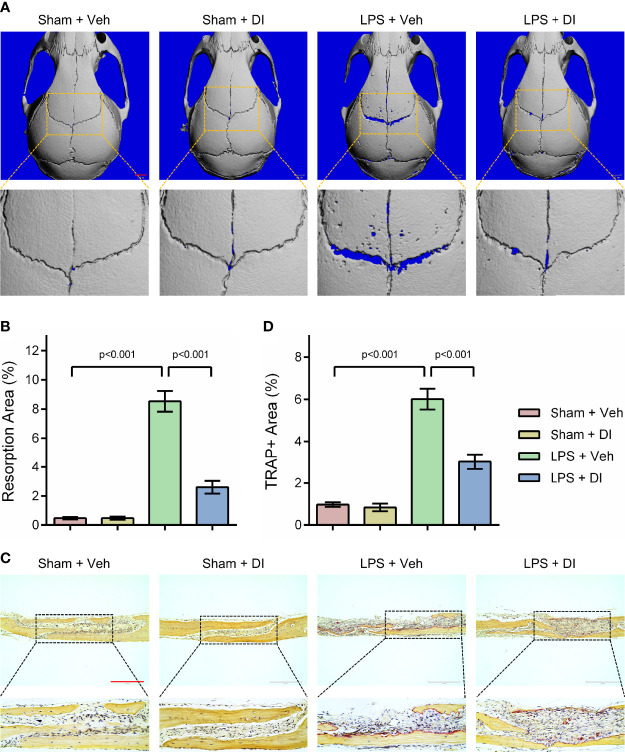
Itaconate attenuates LPS induced inflammatory bone loss *in vivo* **(A, B)** Micro‐CT scanning and subsequent 3D reconstruction of the calvaria bones from the Sham+Veh group, Sham+DI group, LPS+Veh group, and LPS+DI group (scale bar = 1mm). Representative 3D reconstructed images from each group were shown with a blue background. The region of interest was zoomed in and shown below the original 3D reconstructed image, the blue background within the interested area was quantified as a resorption area. **(C)** TRAP staining of calvaria sections from the Sham+Veh group, Sham+DI group, LPS+Veh group, and LPS+DI group (scale bar = 200μm). **(D)** Determination of TRAP (+) area. There were 6 randomly assigned mice in each group. Data are presented as the mean ± SEM of three independent experiments with p value shown in the figure.

Lipopolysaccharide (LPS) produced by bacteria has been identified as the key mediator of chronic inflammation and has been widely used as an inducer for inflammatory bone loss model (27). We tested whether itaconate treatment could rescue LPS induced inflammatory bone loss in the mouse model. LPS was given subcutaneously on the middle cranial suture and itaconate was given through intraperitoneal injection. μCT scanning and reconstructed images of skulls were used to evaluate bone loss severity. We observed significant bone resorption along the middle cranial suture on the skulls from LPS+Veh group, whereas DI treatment effectively rescued bone resorption induced by LPS in LPS+DI group (**Figure 4A**). The percentage of resorption area of skulls were quantified and showed that circulatory addition of itaconate protected inflammatory bone loss induced by LPS *in vivo* (**Figure 4B**). The inhibitory effect of itaconate on inflammatory bone loss was further confirmed by TRAP staining of sectioned skull slides. The results showed that DI treatment decreased the percentage of TRAP+ area and osteoclast formation induced by LPS (**Figures 4C, D**).

### Potential Mechanisms of Inhibitory Effect of Itaconate on Osteoclast

**Figure 5:**
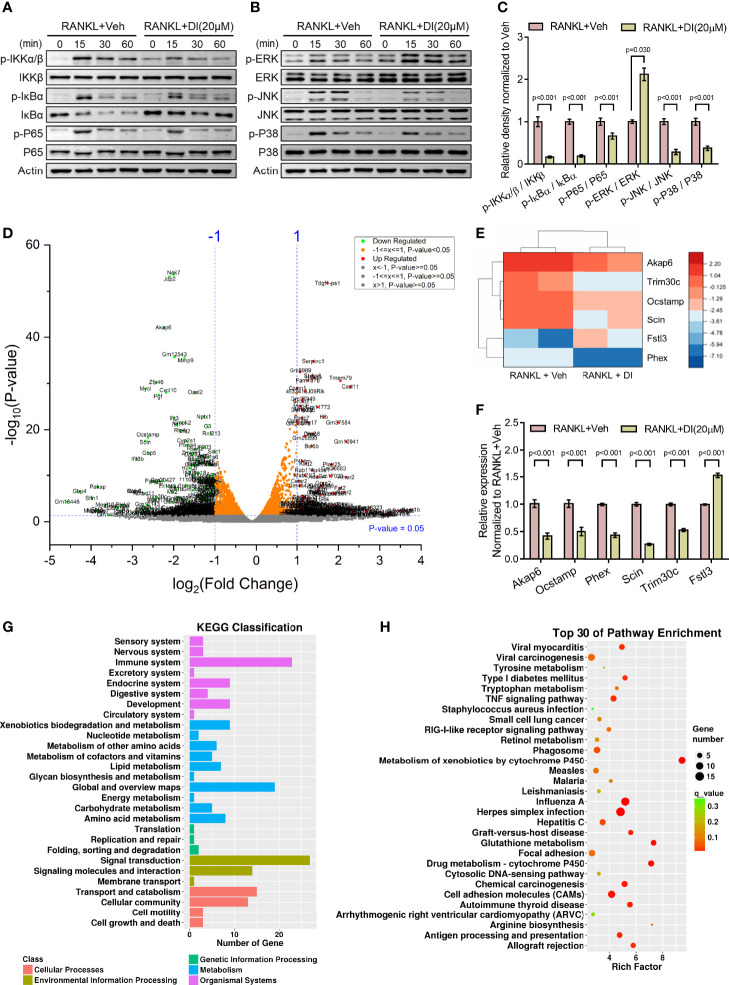
Potential mechanisms of inhibitory effect of itaconate on osteoclast **(A–C)** BMMs were cultured with α-MEM in the absence of FBS for 12 h, and then pretreated with DI (20 μM) or the vehicle for 2 h. Finally, BMMs were stimulated with or without RANKL (100 ng/mL) for the indicated times. Total and phosphorylated protein levels of NF-κB **(A)**, and MAPK **(B)** signaling pathway components were analyzed by Western blotting. Quantification analysis of phosphorylated protein/total protein from **(A, B)** at time point 15 min of RANKL stimulation were presented in **(C)**. **(D)** Volcano plot showing differentially expressed genes upon DI treatment. **(E)** The heatmap of 6 differentially expressed genes which might be involve in skeletal homeostasis, including 5 downregulated genes Akap6, Trim30c, Ocstamp, Scin, Phex and 1 upregulated gene Fstl3. **(F)** The expression of these representative genes was verified by qPCR. **(G)** KEGG classification enrichment analysis of differential genes. **(H)** KEGG pathway enrichment analysis of differential genes. Data are presented as the mean ± SEM of three independent experiments with p value indicated in the figure.

Itaconate has been reported as an anti-inflammation metabolite in macrophages by activating Nrf2 and inhibiting COX2 expression (24, 28). To explore the mechanism underlying itaconate regulated BMMs during osteoclastogenesis, two key signaling pathways related to osteoclast differentiation were assessed by western blot. The results showed that itaconate inhibits the activation of the NF-κB and MAPK pathways by inhibiting phosphorylation of IKKs, IκBα, P65, JNK, and P38, but not ERK protein (**Figures 5A–C**).

RNA-Sequencing of DI or vehicle treated BMMs during osteoclastogenesis was performed to reveal potential mechanisms. Differentially expressed gene assay highlighted significant gene profile alteration upon DI treatment as shown by volcano plot (**Figure 5D**). From the volcano plot, we selected 6 differentially expressed genes which might be involve in skeletal homeostasis and presented the heatmap for these differentially expressed genes, including 5 downregulated genes Akap6, Trim30c, Ocstamp, Scin, Phex and 1 upregulated gene Fstl3 (**Figure 5E**). The expression of these representative genes was then verified by qPCR (**Figure 5F**). KEGG classification enrichment analysis of differential genes indicated that genes related to signal transduction, immune system and metabolism were enriched after DI treatment (**Figure 5G**). KEGG pathway enrichment analysis of differential genes highlighted that Metabolism of xenobiotics by cytochrome P450, Glutathione metabolism and Drug metabolism-cytochrome P450 pathways were heavily involved (**Figure 5H**).

## Discussion

In the present study, we focused on the metabolic remodeling of osteoclast precursor macrophages during aging, and the impact of these changes on osteoclast differentiation and bone homeostasis. We observed that itaconate is accumulated in aged macrophages, indicating an increased basal inflammation level. Itaconate is responsive to RANKL stimulation during osteoclastogenesis in both bone marrow derived primary macrophages and macrophage cell line RAW264.7 cells. Interestingly, the response of itaconate to RANKL stimulation is significantly impaired in aged macrophages. The inhibitory role of itaconate in osteoclast differentiation and activation was confirmed *in vitro*, and the rescue of LPS induced inflammatory bone loss by itaconate was verified *in vivo*. Mechanistically, itaconate treatment impaired NF-κB and MAPK signaling pathway, itaconate induced differentially expressed genes were enriched in KEGG classification of signal transduction and immune system.

IRG1/itaconate metabolic pathway plays a central regulatory role in regulating macrophage activity and links metabolism to immunity (21, 24). Our data revealed a novel IRG1/itaconate expression pattern in young and aged macrophages, which indicate that this metabolic pathway is also involved in the aging process. On the one hand, higher basal level of IRG1/itaconate observed in aged macrophages may be associated with increased basal level of inflammation during aging, which is also known as inflammaging (9, 29). On the other hand, less responsive IRG1/itaconate metabolic pathway upon stimulation in aged macrophages could be associated with age-related immunosenescence (30, 31). Our findings revealed the dichotomous immune status of aged macrophages. However, this phenomenon could also be due to the heterogeneity of aged macrophages which has been observed during efferocytosis (32). Therefore, we anticipate that further characterization of aged macrophages by single cell sequencing will facilitate the understanding of age-related macrophages remodeling.

Recent studies have identified two osteoclast subtypes in bone marrow, the vessel-associated osteoclasts (VAO) which are highly associated with bone endothelial cells to regulate endochondral ossification, and the classical bone-associated osteoclasts (BAO) (33). Osteoclasts are derived from macrophage/monocyte lineage in bone marrow. The bone marrow is responsible for providing supportive microenvironments for hematopoiesis, osteogenesis, angiogenesis, as well as the interactions of these fundamental process to maintain the skeletal homeostasis. Upon aging, inflammation, and other stress factors, the remodeling of bone marrow niches could lead to impaired or imbalance skeletal homeostasis (34–36). The shifting of predominant osteoclast subtype from VAOs in early developing bones to BAOs in ageing bones has been observed during aging (33). In our study, we observed distinct osteoclastogenesis potentials of young and aged bone marrow macrophages, however we didn’t verify the subtype and activity of these osteoclasts. It is possible that young and aged BMMs trend to differentiate to distinct subtype of osteoclasts and exhibit different activities in terms of resorbing cartilage or bone matrix.

Metabolic remodeling has been reported to be essential for osteoclast formation and activity (37, 38). Our data showed that the IRG1/itaconate metabolic pathway is involved in osteoclast formation and activity, supported by up regulated IRG1 expression during osteoclastogenesis. We further tested the impact of itaconate, IRG1 encoded enzyme aconitase decarboxylase produced metabolite, on osteoclast. Interestingly, itaconate exhibited an inhibitory effect on osteoclast differentiation and bone resorption. *In vivo* data showed that itaconate could rescue LPS induced inflammatory bone loss. Given the inhibitory role of itaconate in regulating osteoclast, we propose that increased osteoclastogenesis activity of aged macrophages could be due to impaired IRG1/itaconate response to chronic inflammation.

Dimethyl itaconate (DI) was used in this study to explore the role of endogenous itaconate in regulating osteoclast, as itaconate acid has been considered to be negatively charged polar metabolite which has poor cell permeability. However, this derivative may not recapitulate exactly the same effects of the endogenous itaconate. In a recent comparative study evaluating unmodified itaconate and a panel of commonly used itaconate derivatives, the authors showed that neither dimethyl itaconate (DI), 4-octyl itaconate (4OI) nor 4-monoethyl itaconate (4EI) are converted to intracellular itaconate, while exogenous unmodified itaconic acid can actually enter macrophages. Besides, dimethyl itaconate and 4-octyl itaconate induce a stronger electrophilic stress response and this correlates with their immunosuppressive phenotype (22). The similarities and differences between dimethyl itaconate and endogenous itaconate in terms of regulatory mechanism need to be further addressed. To better justify the role of endogenous itaconate, unmodified itaconate might be a better exogenous itaconate to use in the future.

While other studies have reported metabolic remodeling during osteoclast differentiation (37, 39), to our knowledge this is one of the first studies that has shown metabolites itaconate was involved in inflammaging of skeletal system, and played negative role in the regulation of osteoclast formation and activity.

## Data Availability Statement

The sequencing data presented in the study are deposited in the GEO database, the assigned GEO accession numbers is GSE206723.

## Ethics Statement

The animal study was reviewed and approved by Institutional Animal Care and Use Committee of Tongji Hospital.

## Author Contributions

YW, SL, and LZ conceived the study, performed most of the experiments, analyzed the results, and prepared the manuscript. PC and JL performed experiments. FG, JX, WZ, and AC supervised this study. All authors contributed to the article and approved the submitted version.

## Funding

This project was supported by Grant Number 81902262 and 81672168 from the National Natural Science Foundation of China.

## Conflict of Interest

The authors declare that the research was conducted in the absence of any commercial or financial relationships that could be construed as a potential conflict of interest.

## Publisher’s Note

All claims expressed in this article are solely those of the authors and do not necessarily represent those of their affiliated organizations, or those of the publisher, the editors and the reviewers. Any product that may be evaluated in this article, or claim that may be made by its manufacturer, is not guaranteed or endorsed by the publisher.
